# A technique for lyopreservation of *Clostridium ljungdahlii* in a biocomposite matrix for CO absorption

**DOI:** 10.1371/journal.pone.0180806

**Published:** 2017-07-05

**Authors:** Mark J. Schulte, Jason Solocinski, Mian Wang, Michelle Kovacs, Ryan Kilgore, Quinn Osgood, Lukas Underwood, Michael C. Flickinger, Nilay Chakraborty

**Affiliations:** 1 Department of Chemical and Biomolecular Engineering, North Carolina State University, Raleigh, North Carolina, United States of America; 2 Department of Mechanical Engineering, University of Michigan Dearborn, Dearborn, Michigan, United States of America; 3 Biomanufacturing Training and Education Center, North Carolina State University, Raleigh, North Carolina, United States of America; Ohio State University, UNITED STATES

## Abstract

A system capable of biocatalytic conversion of distributed sources of single carbon gases such as carbon monoxide into hydrocarbons can be highly beneficial for developing commercially viable biotechnology applications in alternative energy. Several anaerobic bacterial strains can be used for such conversion. The anaerobic carbon monoxide-fixing bacteria *Clostridium ljungdahlii* OTA1 is a model CO assimilating microorganism that currently requires cryogenic temperature for storage of the viable strains. If these organisms can be stabilized and concentrated in thin films in advanced porous materials, it will enable development of high gas fraction, biocomposite absorbers with elevated carbon monoxide (CO) mass transfer rate, that require minimal power input and liquid, and demonstrate elevated substrate consumption rate compared to conventional suspended cell bioreactors. We report development of a technique for dry-stabilization of *C*. *ljungdahlii* OTA1 on a paper biocomposite. Bacterial samples coated onto paper were desiccated in the presence of trehalose using convective drying and stored at 4°C. Optimal dryness was ~1g H_2_O per gram of dry weight (g_DW_). CO uptake directly following biocomposite rehydration steadily increases over time indicating immediate cellular metabolic recovery. A high-resolution Raman microspectroscopic hyperspectral imaging technique was employed to spatially quantify the residual moisture content. We have demonstrated for the first time that convectively dried and stored *C*. *ljungdahlii* strains were stabilized in a desiccated state for over 38 days without a loss in CO absorbing reactivity. The Raman hyperspectral imaging technique described here is a non-invasive characterization tool to support development of dry-stabilization techniques for microorganisms on inexpensive porous support materials. The present study successfully extends and implements the principles of dry-stabilization for preservation of strictly anaerobic bacteria as an alternative to lyophilization or spray drying that could enable centralized biocomposite biocatalyst fabrication and decentralized bioprocessing of CO to liquid fuels or chemicals.

## Introduction

Energy efficient stabilization of anaerobic bacteria capable of fixing single carbon gases, such as carbon monoxide (CO), into hydrocarbons can be highly beneficial for developing commercially viable biotechnology applications [1, 2]. While dry-stabilization techniques have been successfully developed for preservation of microbial biocatalysts such as lactic acid bacteria [3] and yeast [4], no such technique has been developed for stabilization of anaerobic microorganisms that can assimilate gaseous carbon compounds other than carbon dioxide. Stabilization of anaerobic bacteria that are capable of assimilating single carbon gases, such as *C*. *ljungdahlii*, and other clostridia, could prove to be highly beneficial in developing bioprocess-oriented large scale gas treatment technologies [5–7].

Dry-stabilization is a biomimetic preservation technology based on the phenomenon of anhydrobiosis—a technique that allows several organisms in nature to survive in the dry state [8]. Organisms from several different phyla, including several land plants, certain fungi, bacteria, several nematodes, tardigrades, rotifers, and brine shrimps can tolerate severe desiccation for extended periods of time [8, 9]. Under such desiccated conditions, the organisms remain in suspended animation with no detectable metabolic activity (cryptobiosis) [10]. Several theories exist as to the molecular mechanisms employed by anhydrobiotic organisms [9, 11]. Of interest is that at such low water levels, bulk water in cells no longer exists and such a condition facilitates development of a low molecular mobility environment where molecular reactions and related degradative reactions are restricted, leading to stabilization [11]. Such vitrified (high viscosity) environments are facilitated by the presence of glass forming carbohydrates such as trehalose [12].

While the basic tenets of the technique of anhydrobiosis have been used in several biotechnological applications including; pharmaceutical formulation development [13, 14], agricultural biologicals [14, 15], food production [15, 16] and storage [16], pest control [17, 18], and biosensors [18], each application has its own optimum processing requirements. In this study, processing parameters are investigated to determine optimal lyo(dry)-processing conditions for an anaerobic microorganism to be used as biocatalysts for large scale bioprocessing applications. A Raman microspectroscopy based non-destructive technique was employed to determine the optimum level of dryness.

Herein we report a technique to rapidly desiccate and store strictly anaerobic bacteria (*C*. *ljungdahlii* OTA1) embedded in a paper biocomposite in the presence of trehalose and demonstrate the post-rehydration recovery of activity by monitoring carbon monoxide assimilation. The development of a dry–stabilization technique for an anaerobic bacteria that traditionally requires cryogenic temperatures for successful storage [19] is a critical step forward in large scale bioprocessing of this type.

In this study, a thin-film coating based approach of embedding the microbial samples in paper based biocomposite was undertaken [5, 20], and a simple convective drying mechanism using inert argon gas purge technique was used for dry-processing the samples. The technique employed here is significantly different and simpler from the conventional drying modalities commonly used in the bioprocessing industry such as lyophilization and spray drying. While lyophilization has been successfully implemented in a multitude of biotechnology applications, the technique requires significant developmental effort, is highly energy intensive, and is most importantly difficult to efficiently scale up [21]. Spray drying is commonly used in the dairy industry [17], but the processing requirement involving higher temperatures and pressure are damaging for the majority of unicellular organisms [21].

*C*. *ljungdahlii* OTA1 samples coated in paper based biocomposite remains in growth limited condition and thus offers a matrix for several operational advantages including; reduced matrix cost, low power requirement during operation, reduced operating liquid requirement, and significantly increased mass transfer rates [5, 20]. However, the non-uniform nature of the paper-based biocomposite offers significant challenges when it comes to developing an optimum drying protocol with accurate spatial estimation of residual moisture content. Random distribution of the paper fibers create a highly non-uniform environment. Therefore, precise high resolution spatial estimation of the residual moisture content is a key factor in ensuring that low molecular mobility is achieved in the desiccated samples. In this study, a combined high resolution Raman microspectroscopy and hyperspectral imaging technique was used along with traditional gravimetric techniques to quantify residual moisture. Use of the Raman microspectroscopy based techniques allowed a detailed investigation of the effect of the presence of residual moisture in a microscale environment of the paper biocomposite. It was found that the optimum lyoprocessing condition identified by post-rehydration reactivity had considerable differences between the bulk moisture estimation and the spectroscopic estimation techniques employed here.

The technique described here can enable development of dry-stabilized paper biocomposite biocatalysts containing very concentrated bacteria capable of operating highly reactive gas contacting modules for purifying gases and recycling carbon. The non-woven paper biocomposite modules developed here could be stored and shipped at non-cryogenic temperatures and rehydrated at the site of gas-processing. This would facilitate the design of a new generation of biocatalysts for gas purification for both continuous and distributed bioprocessing of carbon containing gases with minimal water requirement.

## Materials and methods

### Bacterial strain, media, and growth conditions

Mutant *C*. *ljungdahlii* OTA1 cell pellets were sourced and prepared as previously described [5]. Briefly, cell suspension was transferred to two sterile 50mL centrifuge tubes sealed with silicone gasket caps in an anaerobic chamber. The cells were centrifuged at 4°C for 15 min at 6000x g and then the tubes were moved into the anaerobic chamber. After decanting the supernatant, the pellet was mixed until homogenous. Coating emulsion was prepared by adding cell paste to 1YCM at 30% (v/v) and applied at 6.5 g_DCW_/m^2^. The departures from the previous methods are limited to the reducing agent. For growth in mRCMf, bottles were reduced with 3 mL of 2.5% Cysteine-HCl prepared in anoxic 0.1 N NaOH. The limiting growth media was prepared according to the pH required by mixing the cysteine either in anoxic water, 0.1 N, 0.2 N, or 0.4 N NaOH. In the pellet preparation, the cells were rinsed with 2.5% cysteine-HCl in 0.2 N NaOH.

### Coating of biocomposite paper substrate

3MM chromatography paper (Whatman, 0.34 mm thickness, 130 mm/30 min flow rate) was prepared by cutting into strips (2x13 cm, 335 μm thick), wrapped in aluminum foil, autoclaved for 20 min (dry cycle), and stored in a humidity controlled anaerobic chamber (~3% H_2_ in Ar. 26°C, ~40% relative humidity). All materials were left uncovered in the anaerobic chamber for at least one day to ensure anoxic quality. The coating formulation was prepared directly before use. The 10 cm^2^ coatings were applied to one side of the strip by pipette extrusion of 200μL of coating suspension evenly in the anaerobic chamber.

### Drying, storage, and rehydration method

**Fig 1:**
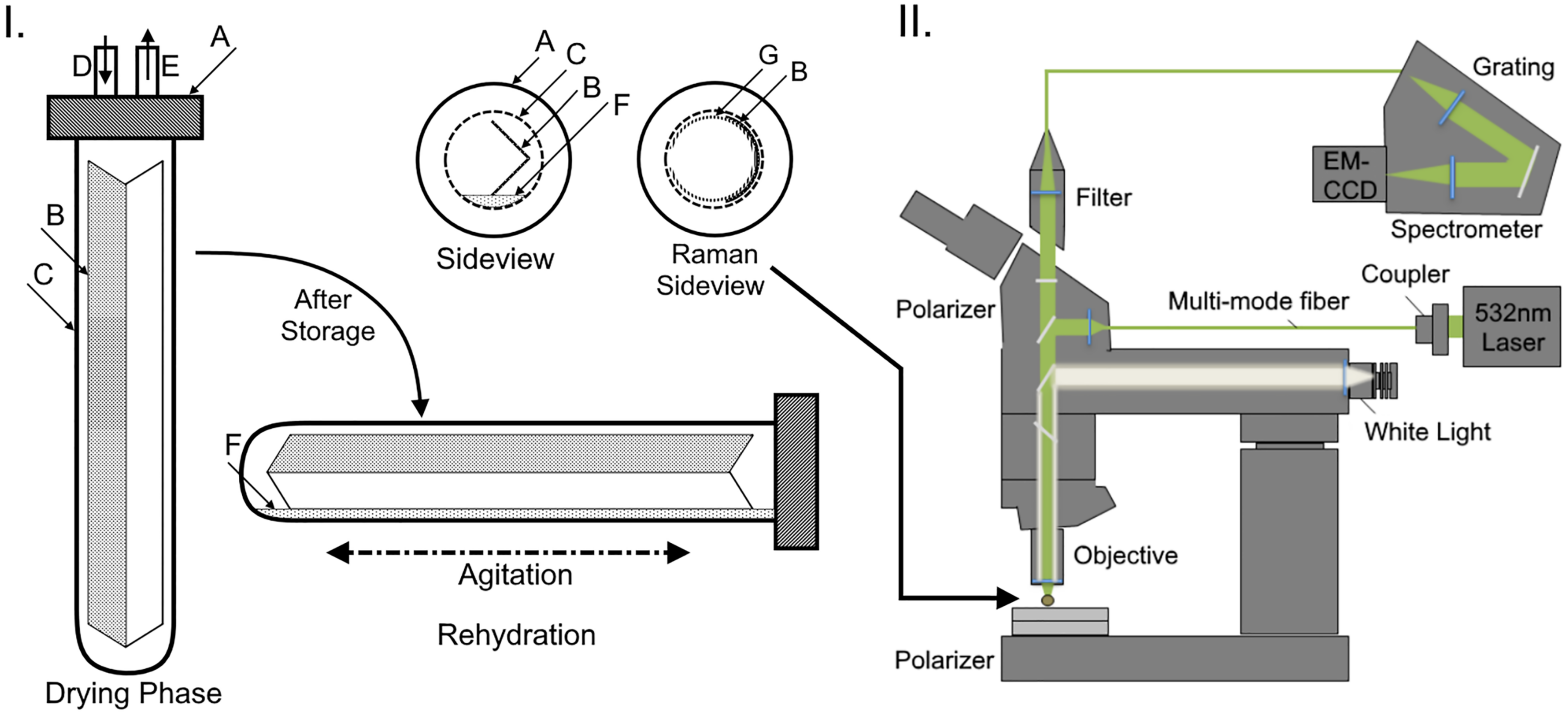
Depiction of convective drying technique and Raman apparatus. **I)** During drying, the upright Balch tube (C), containing the biocomposite paper (B) and stoppered with a rubber gasket (A), was purged with pure argon (D) and was vented of humidified atmosphere (E). After storage, the tubes were rehydrated with growth medium (F) and agitated. The shaded area of the biocomposite is the half which had the biocomposite coating applied. The Raman side view shows how the falcon tube piece (G) was used as a press-lock to keep the paper flush with the tube wall during analysis. **II)** An illustration of the Raman apparatus configuration; laser, polarized microscope, and spectrometer detector.

The freshly prepared biocomposite strips were placed into a Balch tube and sealed with a black butyl rubber stopper (Geo-Microbial Technologies, Inc., Ochelata, OK) in the anaerobic chamber (Fig 1). The tubes were removed from the anaerobic chamber and crimped with aluminum seals. Tubes were flushed with pure argon (Arc3 gases, Raleigh, NC) using two 21G, 1-inch needles with the inlet needle pointing away from the coating surface. After the prescribed time had elapsed, vent needles were removed and the pressurized tube was adjusted to atmospheric pressure. Tubes were then stored in the dark at different temperatures (4, 21, 26 and 37°C). Tubes containing the biocomposite-coated paper strips were rehydrated by adding 4mL of fresh media of the same composition in which the paper was desiccated. The tubes were then flushed with a syngas mixture (45% CO/ 45% H_2_/ 10% N_2_) for 2 min at 20 psi using 21G needles assembly as shown in Fig 1. Tubes were arranged horizontally in a shaker at 37°C, 50 rpm. For determination of gas consumption rates, tubes were sampled with gas tight syringes and analyzed by gas chromatography (GC) as described elsewhere [5]. Briefly; H_2_, O_2_, N_2_, CO, and CO_2_ analysis was performed using an Agilent 7890 gas chromatograph containing a Supelco (Bellefonte, PA) 60/80 mole sieve 5A column (6’ x 1/8” x 2.1 mm SS) and a thermal conductivity detector. Argon was used as the carrier gas. Oven temperature was held constant at 170°C for 1.5 min then increased to 240°C for 3.4 min. The total run time was 5.9 min.

### Evaluation of residual water content

Water content after drying was evaluated gravimetrically in parallel samples (Mettler-Toledo XS64, Columbus, OH). Paper biocomposite samples were weighed (average 0.4769 g ± 0.00173 g SE, n = 18) prior to coating with the *C*. *ljungdahlii* OTA1 cells. Biocomposite biocatalyst strips were convectively dried under anaerobic conditions as described before. Following drying, biocomposites were removed and immediately weighed. To obtain dry weight of the samples, the biocomposites were placed into a vacuum oven overnight at 105°C. They were weighed individually, immediately after coming out of the oven.

### Raman microspectroscopy

Residual moisture content was evaluated by Raman spectroscopy according to the following protocol. Hydrated non-woven paper biocomposite samples were placed into Balch tubes with a plastic ring (3/4 arc cut from a 15mL centrifuge tube) used to keep the paper flush with the interior of the tube. They were then flushed for 0 to 45 min with pure argon and allowed to equilibrate. For the Raman spectroscopic analysis, a customized Raman spectrometer (UHTS 300, WITec Instruments Corp., Germany) and confocal microscope combination was used (Fig 1). A 532 nm solid-state laser was used as the excitation source and the Raman spectra were collected with a sensitive EMCCD camera (Andor Technology, UK), using a 10x objective (Zeiss, Oberkochen, Germany). Point scan arrays were acquired in triplicate for each sample with an integration time of 0.5 s to generate the hyperspectral maps. The samples were collected on the side of the biocomposite substrate that would not have cells applied to it through the glass of the sealed Balch tube. It was assumed that because of the equilibration time and the hydrophilic nature of the paper, that the water was distributed in the matrix of the biocomposite substrate. Hyperspectral images were generated by integrating the CH2 stretching peak (2950 rel. 1/cm), indicating the presence of polymeric fibers in the paper biocomposites, and by integrating the OH stretching peak (3350 rel. 1/cm), which predominately originates from the presence of water molecules in the matrix. The average Raman spectra of the paper biocomposites scan arrays was extracted for respective convective drying conditions.

### Statistical analysis

Data were analyzed with a one-way analysis of variance (ANOVA) on ranks of experimental datasets. Origin Pro 2017 (Northampton, MA) was used for the analyses.

## Results

**Fig 2:**
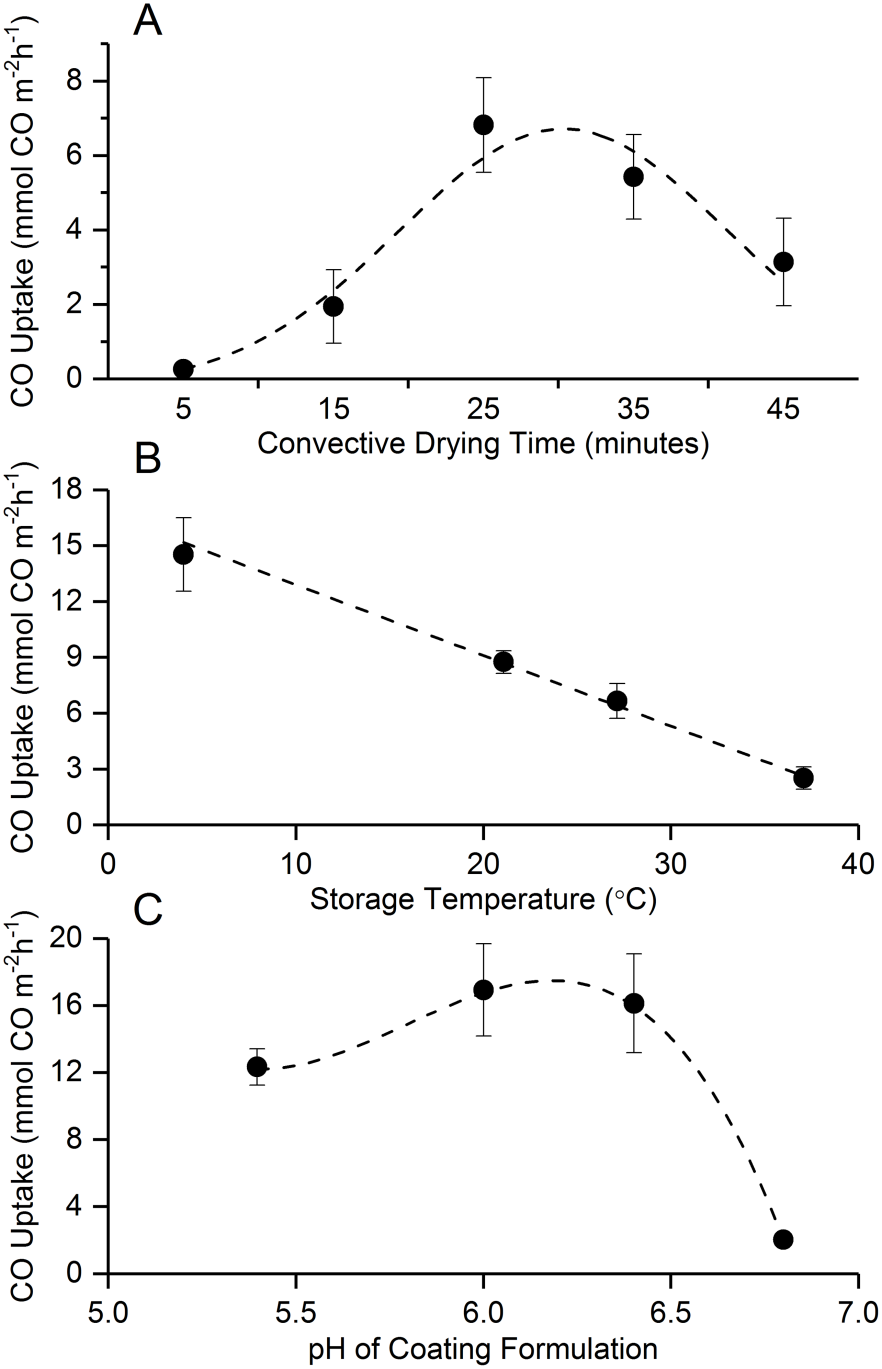
Optimization of bacteria viability. Optimal convective drying conditions were identified using CO uptake rate of the biocatalyst samples measured 75 hours after rehydration using gas chromatography techniques. A) CO uptake rate as a function of convective drying time. Imposed as the dashed line is a Gaussian fit with R^2^ = 0.97733. B) pH of coatings effect upon CO uptake rate. A linear fit was employed, with R^2^ = 0.99687. C) CO uptake efficacy after varying storage temperature. A cubic polynomial fit was applied to show apparent trend of data.

The efficacy of the *C*. *ljungdahlii* OTA1 cells stabilized in the paper biocomposites was evaluated following rehydration by measuring the CO uptake using GC techniques [5]. In an effort to develop an optimized desiccation scheme for these otherwise desiccation- sensitive microorganisms, first the optimum drying time for stabilization of *C*. *ljungdahlii* in paper biocomposites was investigated (Fig 2A). The optimum drying time was determined by desiccating the paper biocomposites containing *C*. *ljungdahlii* OTA1 cells for different time durations. Following desiccation, the samples were rehydrated and 18 hr post-rehydration CO uptake rate was used as an indicator of cellular viability as shown in Fig 2A. It was found that exposure to convective drying conditions for 25 min produced the highest level of post-rehydration CO uptake. Exposure to convective drying conditions both shorter and longer than 25 min yielded significantly diminished CO uptake rates, possibly indicating cellular injury due to rate dependent cumulative osmotic stress.

Next, the optimum storage temperature for the desiccated samples was investigated (Fig 2B). The samples desiccated using the convective drying technique for 25 mins were stored at different temperatures (4, 21, 26, and 37°C) for 75 hours before being rehydrated. A storage temperature below 4°C was not considered due to concerns related to low temperature injury induced by crystallization of residual water [22] and chilling injury [23]. The GC analysis for CO uptake 18 hr post-rehydration indicate that the viability of the cellular samples increases with decreasing storage temperature. Storage at 4°C produces the greatest stabilization outcome. A five-fold increase in CO uptake rate is observed over cells stored at 37°C, with a clear linear trend of increasing CO uptake towards cooler temperatures.

It has been reported that pH can play an important role in modulating performance in bioreactors under anaerobic conditions [24]. It was found that the pH of the coating formulation in the paper biocomposites also plays an important role in modulating the preservation outcome (Fig 2C). It was found that slightly acidic conditions (pH~6.4) improve the CO uptake rate 18 hr post rehydration. pH ranges outside of the physiological norm for clostridia were found to have a detrimental effect on post rehydration CO uptake.

**Fig 3:**
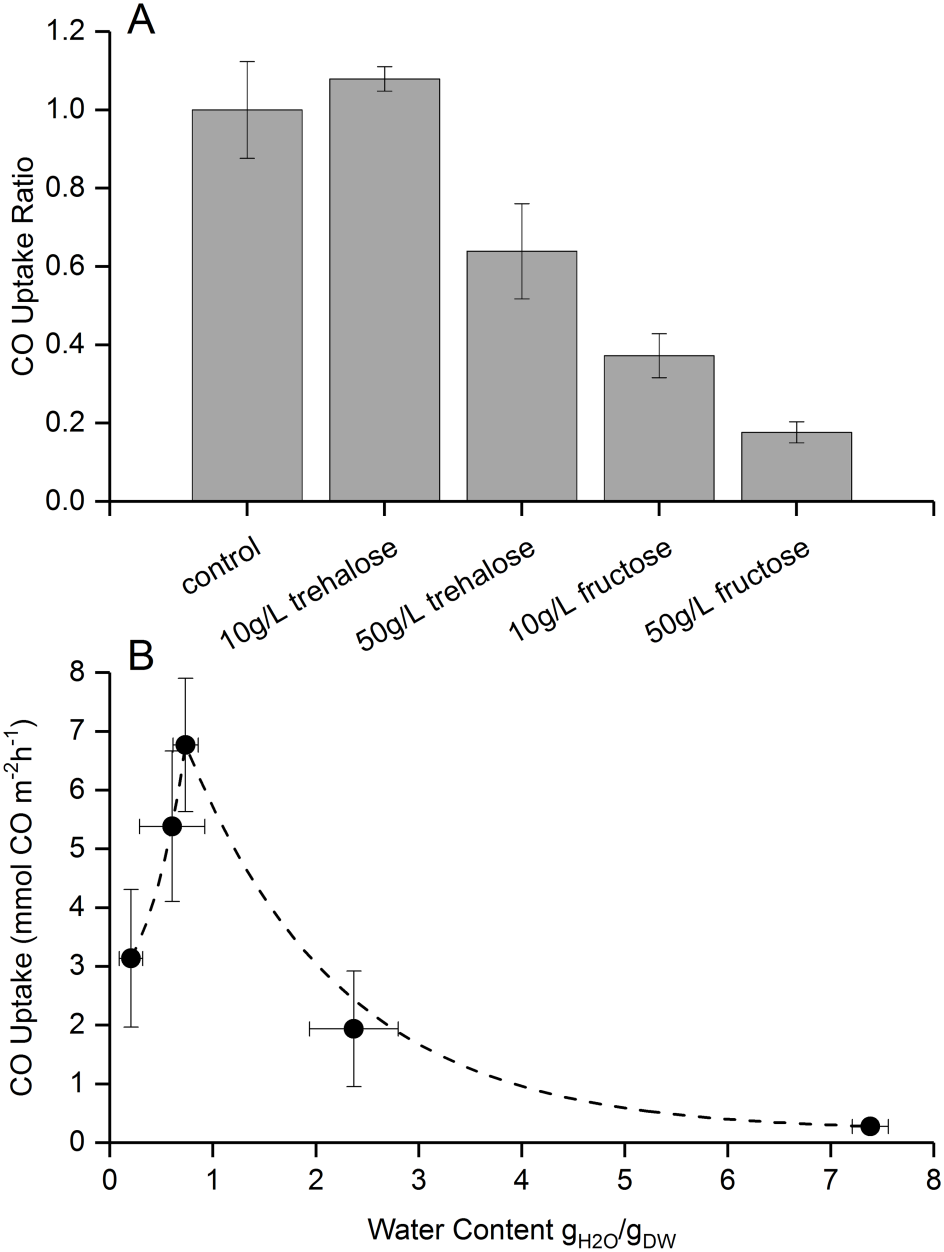
Viability as function of sugar inclusion and residual moisture content. A) Effect of sugar inclusion to coating formulation on viability as measured by CO uptake, normalized to control conditions. B) The gravimetrically determined water content was plotted against the corresponding CO uptake rate of the dried biocatalyst samples. A steady increase in viability up to ~1 g_H2O_/ g_DW_ was noticed which is followed by a marked decrease in the viability response. The dashed line, an exponential growth decay fit, shows this trend.

When designing a protective formulation for lyoprocessing desiccated cellular samples, the effect of the presence of disaccharides such as trehalose has been well studied [25]. However, disaccharide amounts in each formulation needs to be optimized as increased trehalose concentration has been shown to cause cellular injury in mammalian cells [26]. It was found that while the addition of trehalose (10g/L) to the coating formulation on paper biocomposites during desiccated storage *C*. *ljungdahlii* OTA1 did help in reducing the variability in CO uptake outcome in dry stabilized samples in post-rehydration condition, excess of trehalose reduces cellular viability (Fig 3A). However, addition of, a ketonic monosaccharide (fructose) reduced cellular viability in desiccated conditions.

Fig 3B indicates the optimal dryness of the biocomposites. During desiccated storage conditions, a dryness of ~1 g_H2O_ per gram of dry weight (g_DW_) is ideal for cellular viability. The optimal dryness for desiccated storage appears to have a narrow range and the cellular viability quickly decreases in both directions from the optimum.

**Fig 4:**
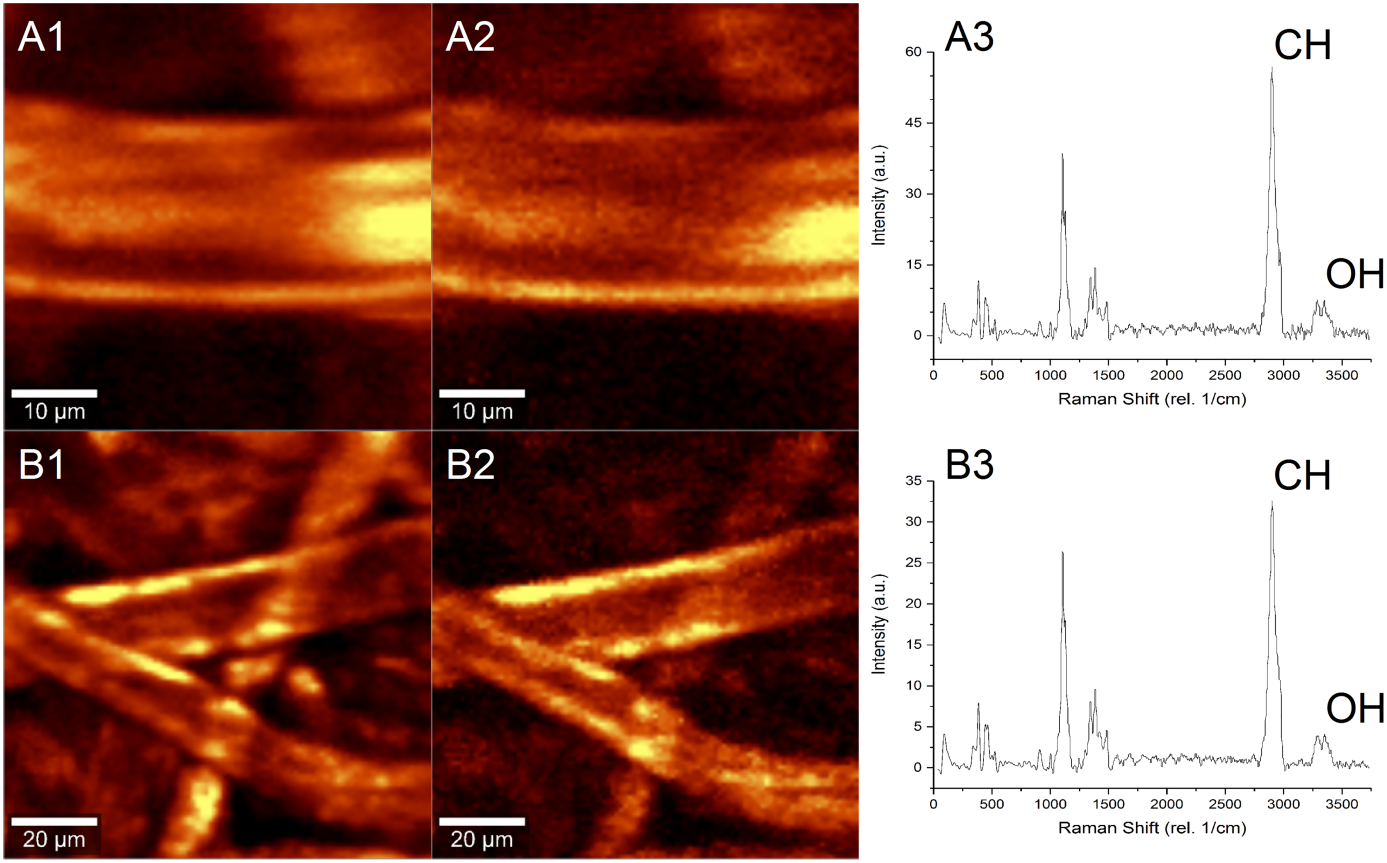
Spatial moisture content using Raman spectroscopy. In the panel, (A) and (B) are 5 min and 35 min convectively dried paper samples, respectively. (1) represents integration on the CH2 stretching peak ~2950 rel. 1/cm, (2) represents integration on OH stretching band ~3350 rel. 1/cm, and (3) is the corresponding averaged Raman spectra for the entire hyperspectral map.

**Fig 5:**
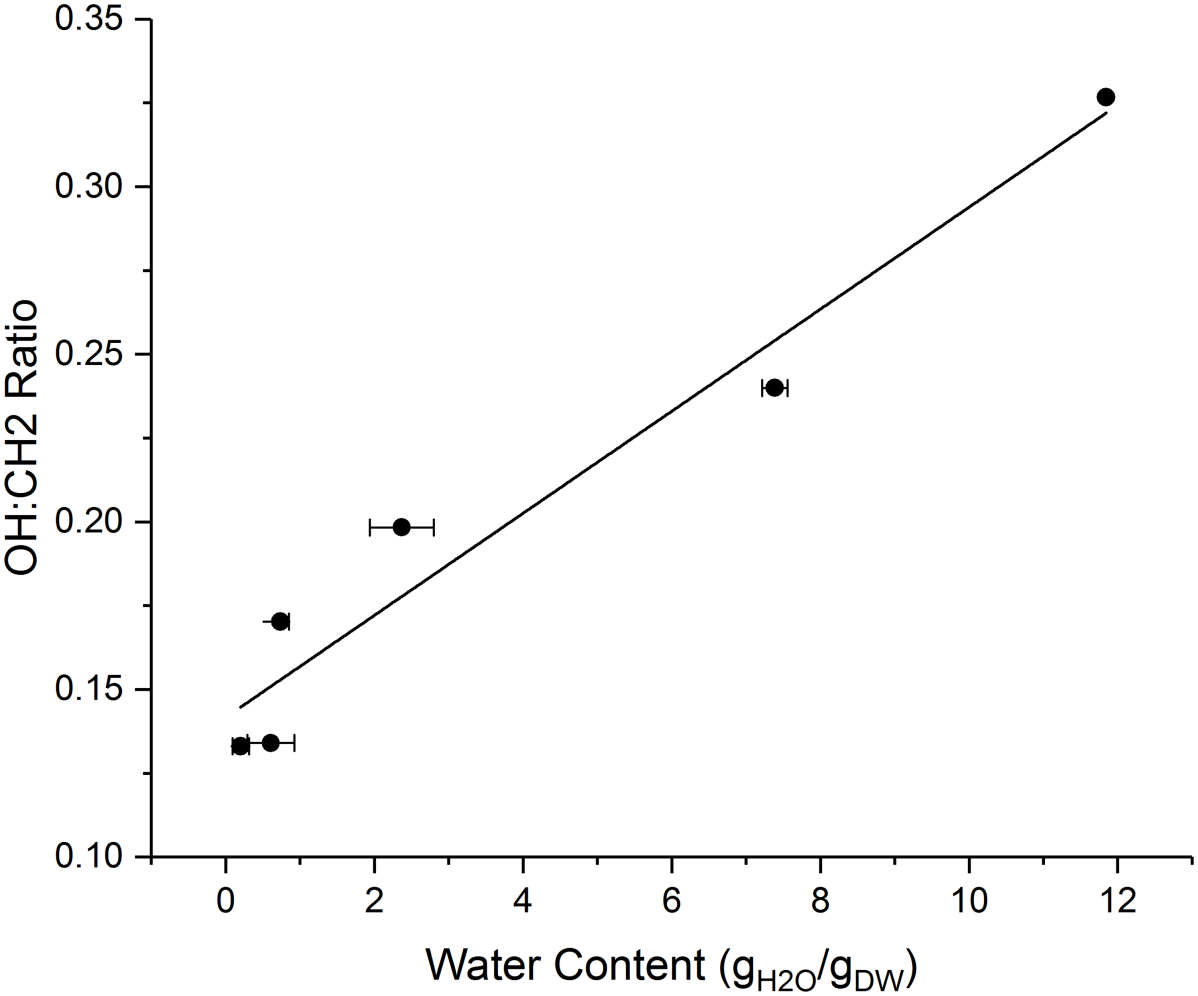
Comparison of gravimetric and spectrally acquired water content. Raman samples were scanned on convectively dried abiotic 3MM paper. Maximum peak intensity ratios of 3350 and 2950 wavenumbers were correlated to gravimetrically obtained total water content of similar paper samples. Values were obtained for samples dried at equal convective drying times. Linear regression fitting shows a strong correlation (R^2^ = 0.95) between the two water content acquisition methods used.

The Raman microspectroscopic analysis of the dried paper biocomposites are shown in Fig 4. Representative hyperspectral images of the paper biocomposites desiccated to different convective drying times—5 min (Figs A1, A2, and A3) and 35 min (Figs B1, B2, and B3) are indicated. Hyperspectral images A1 and B1 are generated by integrating the CH2 stretching peak (2950 rel. 1/cm), while the images A2 and B2 are generated by integrating the OH stretching peak (3350 rel. 1/cm). Fig panels A3 and B3 indicate the average Raman spectra of the paper biocomposites for respective convective drying conditions. The difference in peak intensities in OH stretching peaks in Figs A3 and B3 indicate loss of water molecules from the biocomposite samples due to the increase in convective drying time. Analysis of the spectral results shown are total area averaged, which internally normalizes output spectra to both dimensional area and fiber-to-void ratio which cannot be fully avoided with the paper biocomposites. In essence, a ratio of the OH to CH2 stretching peak intensities indicate the normalized water content per average area of the paper biocomposite and is comparable to the residual water content of the desiccated samples. A direct comparison of the residual water content measured using gravimetric technique with the OH:CH2 ratio indicate a strong linear correlation between the two methods of residual moisture evaluation (Fig 5).

**Fig 6:**
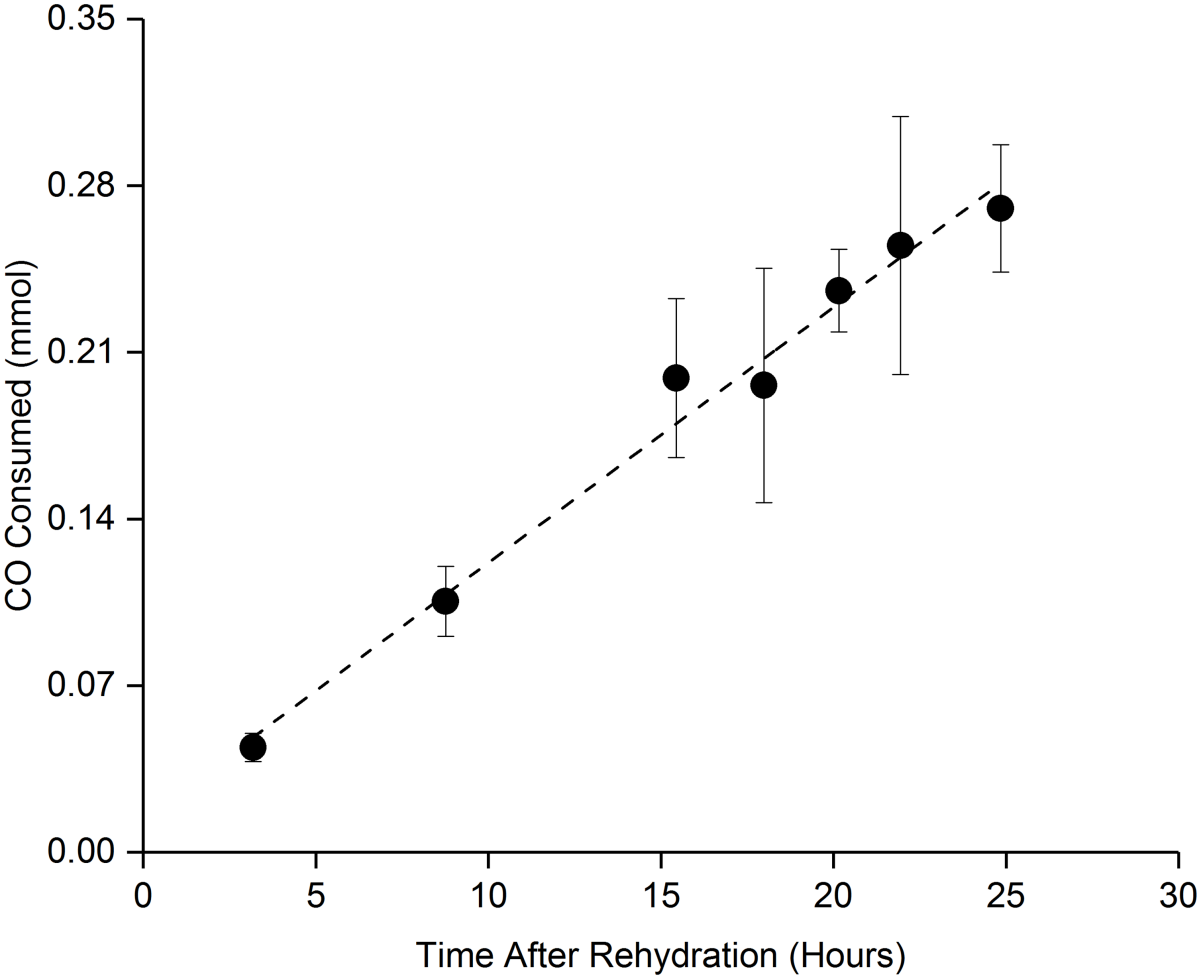
Post rehydration metabolic recovery. Recovery of bacteria metabolic function as measured by CO consumption after rehydration for a 24 hour period. Demonstrates linear increase in metabolism almost immediately after rehydration.

Fig 6 indicates the recovery of the CO uptake activity following rehydration of samples desiccated for 25 min under optimal convective drying conditions. It is interesting to note that the CO uptake starts increasing almost immediately following rehydration and steadily increases over time indicating continuous cellular activity of the dry stabilized *C*. *ljungdahlii* OTA1 cells.

**Fig 7:**
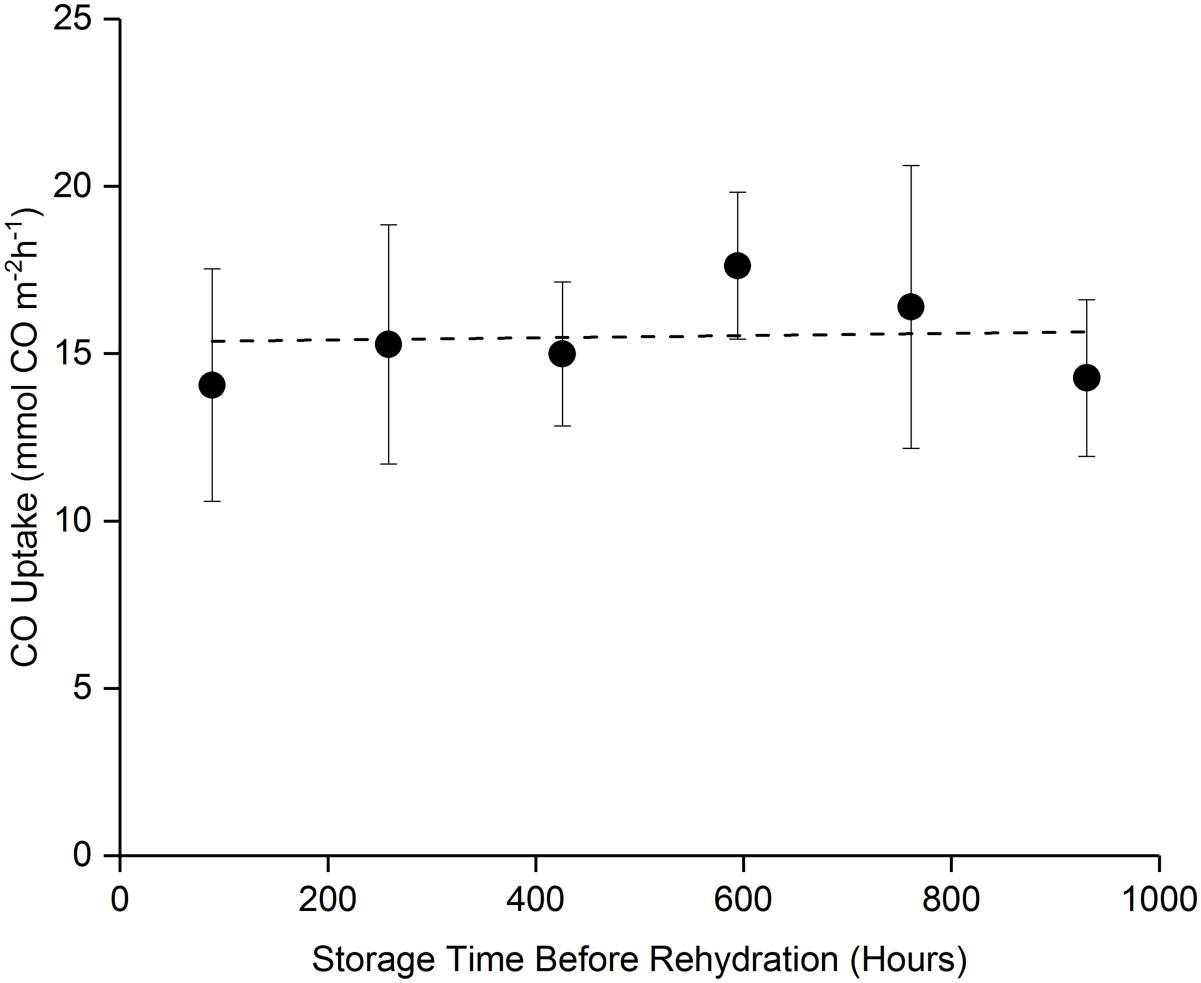
Storage time effect on viability. There was no degradation of bacteria CO uptake rate in the dry state for up to 38 days prior to rehydration. Samples were processed and stored at optimal conditions.

Fig 7 displays a longer time storage stability study performed, aimed at understanding the effect of storage time in the desiccated condition on post-rehydration viability. Remarkably, *C*. *ljungdahlii* OTA1 cells in stored in desiccated conditions (average dryness of ~1 g_H2O_ per g_DW_) at 4°C for over 900 hours (~38 days) without a significant drop in their post-rehydration CO uptake abilities (n = 3; p>0.05). In each case, the CO uptake rates were analyzed 18 hours following rehydration.

Finally, to independently confirm cellular viability and growth potential of dry-stabilized *C*. *ljungdahlii* OTA1 cells in paper biocomposites, a 3 cm^2^ section of the coated paper was collected after the *C*. *ljungdahlii* OTA1 samples were dry stabilized for 38 days as shown in Fig 7 and submerged in an appropriate growth media in anaerobic conditions. The tubes were sealed and incubated at 37°C while being placed on a shaker at 100 rpm. Following 72 hr of incubation, the optical density of the samples was measured at a wavelength of 600 nm (OD_600_) to quantify growth rates. Readings of ~1 were obtained, demonstrating an average growth rate of 0.053 h^-1^. When non-desiccated samples of *C*. *ljungdahlii* OTA1 were incubated under similar conditions, an average growth rate of 0.10 h^-1^ was obtained.

To confirm that the results were not unique to strain OTA1, *C*. *ljungdahlii* WT-55383 acquired from the American Type Culture Collection was also evaluated under similar conditions. A comparable drying and storage experiment was performed and this experiment demonstrated an approximate 20% recovery of wet reactivity (data not shown). However, the wet reactivity of WT was only 20% of OTA1 under similar conditions.

## Discussion

We have demonstrated, for the first time, dry storage of *C*. *ljungdahlii* by convective drying using argon for over 38 days without any loss in CO absorbing reactivity. The extent of desiccation of the cell samples have a direct relationship with the cellular viability. The injury may be due to over-desiccation effects which leads to permanent loss of viability. The optimal length of convective drying was determined to be approximately 25 minutes with further drying toward the glassy regime only decreasing reactivity (Fig 1). Significantly diminished CO uptake rates following rehydration for convective drying conditions shorter and longer than 25 min possibly indicates significant cellular injury. However, there is a strong possibility that the nature of the injury for the longer duration of drying is different from shorter duration of drying. For shorter duration of drying, the injury may come from the accumulation of osmotic stress [27], whereas at a longer duration of drying, the injury may be due to over-desiccation effects. The pH of the media for drying and rehydration was found to not have a significant effect if it was within the normal growth range. Reducing the storage temperature to 4°C increased reactivity after rehydration indicating that some intercellular biochemical reactions likely remain active following desiccation to ~1 g_H2O_/g_DW_ and that these reactions lead to cell death.

When it comes to dry-stabilization of prokaryotes in general, several techniques including freeze-drying [25, 28, 29] and spray drying [12, 30–33] have been used. While freeze-drying is the most frequently used technique for dehydration of propionibacteria [34], implementing the technique is time consuming and costly as it requires careful excipient development and process optimization. In comparison, spray drying has significantly lower development cost. However, the possibility of physical damage due to high pressure and temperature environments during spray drying is a concern [35]. Furthermore, both the techniques require optimization to be developed for anaerobic conditions to be developed for dry-stabilization of anaerobic bacterial cells. Herein a simple anaerobic convective drying-based technique has been developed for stabilization of *C*. *ljungdahlii* OTA1 in a bioreactor format.

As dry-stabilization relies on creation of a highly viscous environment at the cellular level, a glass-forming carbohydrate trehalose has been used in the coating formulation for desiccating the samples. Trehalose has been shown to be effective in creating a highly viscous environment when present in the desiccating matrix [25]. The ability to survive such extreme dehydration conditions has been shown to correlate with the accumulation of large amounts (as much as 60% of their dry weight) of certain intracellular sugars like trehalose, sucrose, etc. [8]. Although there is considerable debate about the exact mechanism(s) of the protective role of sugar, the current state of knowledge has two main postulated mechanisms; (1) stable glass formation of sugar solutions at high temperatures, and (2) stabilization of biological membranes and proteins by direct interaction of sugars with polar residues through hydrogen bonding called the “water-replacement hypothesis” [9]. In this study, the presence of extracellular trehalose significantly reduces the variation in dry-stabilization outcome (Fig 3A).

Due to its role as lyoprotectant, trehalose has been at the center of focus in ambient temperature biostabilization efforts [36]. There is overwhelming evidence that sugars such as disaccharides need to be present on both sides of the plasma membrane in order to provide protection against the damaging effects of desiccation at cellular level [37–39]. While most of the work done in biostabilization at desiccated state involve use of mammalian cells, there is considerable scope to translate the ambient temperature stabilization technologies for dry stabilization of commercially relevant anaerobic bacteria. Biological studies with model membrane systems indicate that to realize the full potential of the lyo-protective effect of trehalose, it needs to be present on both sides of the membrane [36,37]. Studies by Chen et al. [38] and Acker et al. [39] indicate that to recover reasonable membrane integrity of the cells after drying below 5% of moisture content, the intracellular concentration of trehalose has to be more than 200 mM. In fact, most anhydrobiotic organisms have concentrations of internal sugars that range from 20 to 50% of the dry weight of the organism [36], or roughly 0.2 to 0.5 M trehalose before drying. Arguably such high internal concentrations of sugar help in creating a glassy environment inside the cell, retarding the degradative chemical reactions that lead to loss of viability. *C*. *ljungdahlii* OTA1 does not have a native trehalose transporter and the strategies discussed above for trehalose mediated stabilization developed for mammalian cells can be possibly implemented.

This current body of work shows the potential for fabrication of reactive dry-stabilized *Clostridial* biocomposite biocatalyst modules for distributed processing of gases using a microscopic Raman characterization method for rapid determination of residual water content. Optimal drying parameters for *C*. *ljungdahlii* were investigated as a function of metabolic output. Storage for up to 38 days was found to have negligible effect on bacterial viability, which are immediately reactive upon rehydration. By gravimetric and spectroscopic techniques, optimal water content was found to be ~1g_H2O_/g_DW_. Without the need for an inoculum seed train to scale up cell concentrations at every gas processing site, modules could be fabricated at a central location and transported dry to a gas-generating point source for recycling gaseous carbon. With many modules running in parallel, true continuous gas bioprocessing could be achieved by replacing spent modules with freshly rehydrated modules stored dry until needed. This concept could be extended beyond CO to other single carbon or VOC (volatile organic compounds) assimilating microorganisms for recycling gaseous carbon into chemicals and liquid fuels.
